# Tigecycline treatment of infection caused by KPC-producing *Escherichia coli* in a pediatric patient

**DOI:** 10.1186/1476-0711-12-19

**Published:** 2013-08-13

**Authors:** Xiaoxing Du, Ying Fu, Yunsong Yu

**Affiliations:** 1 Department of Infectious Diseases, Sir Run Run Shaw Hospital, College of Medicine, Zhejiang University, Hangzhou, Zhejiang, China

**Keywords:** Tigecycline, Treatment, KPC, Pediatric patient

## Abstract

Tigecycline shows great antimicrobial activity against both Gram-positive and Gram-negative bacteria, and has been considered to be an appropriate choice in controlling infection caused by multi-drug resistant (MDR) pathogens, such as carbapenemase-producing *Enterobacteriaceae* (CPE). Although many clinical trials evaluate the efficacy and safety of tigecycline on adults, rare reports recommend tigecycline to treat pediatric patient. In this study, we presented a clinical case with tigecycline as an anti-infectious agent on a 14-year-old child who was suffering from infection of intraperitoneal abscess caused by *Klebsiella pneumoniae* carbapenemases (KPC)-producing *Escherichia coli* with extreme drug resistant profile. By accessing the clinical outcome and efficacy of the patient, and the side effects of tigecycline, our research explored the documented experience of tigecycline on controlling infection caused by CPE isolate in children.

## Introduction

Carbapenems have been considered to be the last-line antimicrobial agents to against multiple drug resistant (MDR) Gram-negative bacteria for a long time. While recently, the emergence of carbapenemase-producing *Enterobacteriaceae* (CPE) has been reported worldwide increasingly, which has raised global concern for those isolates shows hydrolytic activities to multiple antibiotics including carbapenems [1-3]. There are many carbapenemases involved in CPE isolates, in which *Klebsiella pneumoniae* carbapenemases (KPCs) plays a vital role [1-3]. Although the susceptibility data in vitro indicate that colistin, tigecycline, and fosfomycin are effective to CPE isolates, the optimal treatment choice hasn’t reached agreement yet [2]. Among the above three selectable agents, tigecycline, a semi-synthetic derivative of minocycline and a member of the tetracycline family, shows superiority due to a wider anti-bacterial spectrum especially to MDR pathogens and better tissue permeability particularly to complicated intra-abdominal infection (cIAI) [4,5]. In practice, tigecycline has been approved to treat infection caused by MDR Gram-negative pathogen, especially for the care of critically ill patients [6]. However, it is still not been recommended to be used in pediatric patients, not mention to treat infection caused by *bla*_KPC_-positive isolates. We reported here a documented case about a 14-year-old child who was infected by KPC-producing *Escherichia coli* and was treated with tigecycline.

## Case report

On September 13, 2012, a 14-year-old Chinese patient was transferred to our hospital (Sir Run Run Shaw Hospital, Hangzhou, Zhejiang province, China) due to postoperative intraperitoneal abscess infection of appendicitis.

Three weeks ago (August 25, 2012), the patient was admitted to a local hospital of Hangzhou for vague abdominal pain of the right lower quadrant for one day. The pain was aggravated while walking, but was relieved when lying with knees bent. Total leukocyte count was 11.5 × 10^9^/L with 63.7% neutrophils, his hemoglobin was 13.2 g/dL, and platelet count was 229 × 10^9^/L. The clinical diagnosis of acute appendicitis was confirmed by the B-ultrasonography. Then appendectomy was conducted immediately. While just after the operation, the patient developed severe pain at incision site with frequent chills and fever (over 39°C). The complete blood count (CBC) results indicated that the leukocyte count increased to 14.3 × 10^9^/L with 91.5% neutrophils, and C-reactive protein (CRP) level arrived to 523 mg/L. The patient received aztreonam (1 g q12h) and etimicin sulfate (0.2 g qd) to control postoperative infection for two days, while he persisted with high fever and further developed to lower back pain, accompanied with nausea, vomiting (two to three times per day) and diarrhea (six to seven times per day). Considering the anti-infective effect unsatisfied, moxalactam (latamoxef, 1 g q12h) was prescribed to the patient on the forth hospital day. At this time, computed tomography (CT) scan was conducted, which indicated seroperitoneum, pneumatosis, swelling of soft tissue near right psoas, right kidney enlargement and pleural effusion. Leukocyte count was still high as 12.3 × 10^9^/L with 78.3% neutrophils, and CRP was 96.1 mg/L. Antibiotic treatment was then switched to imipenem (0.5 g q8h) on the fifth hospital day. The temperature could be controlled within 37.5°C to 38°C during the usage of imipenem. Two weeks later, the patient deteriorated to incision suppurates and was transferred to our hospital for further treatment.

Physical examination showed that abdomen was soft and flat, tenderness and rebound tenderness were positive around the incision site. A 3cm-long incision in the right lower quadrant was swelling and purulent. Percussion pain of the right renal region was positive. The leukocyte count was 10.0 × 10^9^/L with 6.5% neutrophils, 70.7% lymphocytes, and 21% monocytes. The hemoglobin, platelets, CRP and procalcitonin (PCT) were 10.7 g/dL, 117 × 10^9^/L, 116.7 mg/L and 2.3 ng/ml, respectively. CT scan showed abscesses spread to right paracolic sulcus, iliaca fossa and liver. On September 14 (the second hospital day in our hospital), the patient’s condition was suddenly worsened, with blood pressure and SaO_2_ down to 70/40 mmHg and 90%, heart rate and respiratory rate up to 130 beats and 25 per minute, which suggested sepsis shock happened. The patient received the second abdominal surgery immediately to repair intestinal fistula. Two *E. coli* isolates were isolated from continual abdominal effusion culture, both of which were pan-resistant to antimicrobial agents by VITEK 2 system, but susceptible to tigecycline, colistin and fosfomycin with MIC 0.25 mg/L, 0.5 mg/L and 16 mg/L using E-test method, respectively (Table 1). According to the susceptibility result, it seemed that only three antibiotics could be used in this case, while colistin is unavailable in China mainland nowadays. It is highly recommended that fosfomycin be used in combination with other agents for the treatment of infections with CPE to prevent the emergence of resistance to this agent [7], so the combination of tigecycline (first dose of 100 mg, then 50 mg q12h, according to 1 mg/kg body weight) and intravenous fosfomycin (4 g q12h) was given to the patient. But fosfomycin had to be stopped in the next day due to its adverse reaction (serious vomiting). Six days after the usage of tigecycline, a *Enterobacter cloacae* isolate resistant to tigecycline with MIC 4 mg/L but susceptible to most β-lactam antibiotics was separated (Table 1), then the antibiotic treatment was switched to cefepime (2 g q12h). Leukocyte count decreased to 8.1 × 10^9^/L with 34.1% neutrophils, 39% lymphocytes, and 26.5% monocytes, and CRP to 9 mg/L. On the 13th hospitalization day in our hospital, the patient was improved greatly and then discharged.

**Table 1:** The susceptibility results

**Isolates**	**TZP**	**AMK**	**CRO**	**FEP**	**GEN**	**CPS**	**ETP**	**IPM**	**MEM**	**TGC**	**CST**	**FOM**
*E. coli* (isolate 1 and 2)	≥128	≥64	≥64	64	≥16	96	≥8	≥16	8	0.25	0.5	16
*E. colace* (isolate 3)	≤4	2	≤1	≤1	≤1	0.19	≤0.5	2	0.064	4	24	8

Note: TZP, piperacillin/tazobactam; AMK, amikacin; CRO, ceftriaxone; FEP, cefepime; GEN, gentamicin; CPS, cefoperazone/sulbactam; ETP, ertapenem; IPM, imipenem; MEM, meropenem; TGC, tigecycline; CST, colistin; FOM, fosfomycin.

Genes associated with carbapenems resistance were detected on the above two *E. coli* isolates, as previous reported, which showed both of them carried *bla*_KPC-2_ and *bla*_CTX-M-24_ gene [8,9].

## Discussion

KPC-producing *Enterobacteriaceae*, as one of MDR pathogens associated to healthcare-associated infection (HCAI) with high mortality, has raised global concern for the outbreaks in northeast of USA, Israel and the east China in recent years [1]. *Enterobacteriaceae* isolates carrying KPCs enzyme exhibit hydrolyzing-activity to wide spectrum of β-lactams, including penicillins, cephalosporins, aztreonam, and even carbapenems. Seriously, most of them usually co-harbor other resistant genes mediating resistance to multiple classes of antibiotics such as fluoroquinolones or aminoglycosides [3], which leads to MDR events. When CPE isolates evolve to MDR, clinical therapy would be immersed to serious situation. The combination of different antibiotics with synergistic mechanisms of action not only may be useful for the management of multidrug-resistant Gram-negative infections but also can lessen the chance of resistance development.

Reports have given some suggestions on combination schemes to CPE isolates [10]. Based on the synergic effect between third generation cephalosporins and amoxicillin/clavulanic acid to KPC producers in vitro, some experts consider cephalosposins like cefepime in combination with amoxicillin/clavulanic acid could produce well effect, but it is still lack of clinical evidence [11]. Besides, colistin, tigecycline and fosfomycin also show pretty well susceptibility to CPE in vitro. Combination therapy based on tigecycline or colistin is considered to be one of the other appropriate choice [12,13]. To those isolates with lower carbapenems MICs, combination based on carbapenems would also be effective. In our case, owning to the very slight synergic effect of cefepime in combination with amoxilin/ clavulanic acid observed in vitro, colistin being unavailable in China mainland and high level of carbapenems MICs (IMP ≥ 16), tigecycline combined with fosfomycin seems to be our unique treatment option.

The activity of tigecycline in vitro has been recognized, and in vivo both the efficiency and the security of tigecycline are confirmed supportive, especially in community-acquired pneumonia (CAP), cIAI and complicated skin and skin structure infections (cSSSI) [14], but it has still not been recommended for the therapy on the pediatric population, even reports are rare. Among those rare reports, tigecycline was used to treat meningitis combined with bacteremia caused by vancomycin-resistant *Enterococcus faecium* and central venous catheter infection caused by MDR *Corynebacter iumjeikeium*, respectively [15,16]. In 2012, Halil Özdemir reported a nine-year-old girl with acute myeloid leukemia who was treated successfully with tigecycline due to multidrug-resistant *E. coli* bacteremia [17]. Although some researches have explored for the treatment of infection involved by KPC-producing *Enterobacteriaceae* with tigecycline, the optimal therapy plan is yet to be established for the lack of the available clinical evidence in children reports.

In this case, we took tigecycline combined with fosfomycin as treatment option. The recommended dosage of tigecycline is calculated with 1.2 mg/kg q12h (no more than 50 mg) in children. During the therapy, the patient developed vomiting, which it was thought to be related to fosfomycin at first, and hence fosfomycin was stopped. But this situation was not completely relieved until tigecycline was stopped. Therefore, it was very possible that the vomiting was related to tigecycline. It has been reported of the side-effect of vomiting involved by tigecycline that the phase II clinical trials revealed 50% children suffered from nausea and vomiting, majority of which were reported as mild to moderate in toxicity of tigecycline [18,19]. During the usage of tigecycline in our patient, an *E. colace* resistant to tigecycline was emerging, which presents the limitation of tigecycline. There several researches have reported monotherapy of tigecycline is restricted for treating infections, which involve with inducing resistant events and higher mortality [20-22]. Therefore, tigecycline may be used only as salvage therapy in critically ill patients, and if it is used for pediatric patients, more concerns should be taken because of possible side effects.

## Conclusion

Owing to the limited evidence of tigecycline being used in pediatric patients, we did some documented experience to use monotherapy with tigecycline to treat KPC-producing *Enterobacteriaceae* infection in children. Our results confirmed the effect of tigecycline to control infection caused be CPE, while drug induced side effects like vomiting and inducing resistance were also observed during the therapy. Although infection was controlled successfully in this case, it is hard to evaluate the efficacy and the safety of tigecycline in treating pediatric patients, more concerns should be taken because of possible side effects.

## Consent

Written informed consent was obtained from the patient for publication of this Case report and any accompanying images. A copy of the written consent is available for review by the Editor-in-Chief of this journal.

## Abbreviations

MDR: multi-drug resistant; CPE: carbapenemase-producing *Enterobacteriaceae*; KPCs: *Klebsiella pneumoniae* carbapenemases; cIAI: complicated intra-abdominal infection; CBC: complete blood count; CRP: C-reactive protein; CT: computed tomography; PCT: procalcitonin; HCAI: healthcare-associated infection; CAP: community-acquired pneumonia; cSSSI: complicated skin and skin structure infections.

## Competing interests

All authors do not have any potential, perceived, or real conflict of interest.

## Authors' contributions

All authors made substantial contributions to the acquisition of data. DX assisted in data gathering and FY helped in drafting the manuscript. All authors read and approved the final manuscript prior to publication.
